# *ZMYND10*, an epigenetically regulated tumor suppressor, exerts tumor-suppressive functions via miR145-5p/*NEDD9* axis in breast cancer

**DOI:** 10.1186/s13148-019-0785-z

**Published:** 2019-12-04

**Authors:** Yan Wang, Liangying Dan, Qianqian Li, Lili Li, Lan Zhong, Bianfei Shao, Fang Yu, Sanxiu He, Shaorong Tian, Jin He, Qian Xiao, Thomas C. Putti, Xiaoqian He, Yixiao Feng, Yong Lin, Tingxiu Xiang

**Affiliations:** 1 Key Laboratory of Molecular Oncology and Epigenetics, The First Affiliated Hospital of Chongqing Medical University, Chongqing, China; 2 The People’s Hospital of Tongliang District, Chongqing, China; 3 Cancer Epigenetics Laboratory, Department of Clinical Oncology, State Key Laboratory of Translational Oncology, Sir YK Pao Center for Cancer, Li Ka Shing Institute of Health Sciences, The Chinese University of Hong Kong, Hong Kong, Hong Kong; 4 Department of Pathology, Yong Loo Lin School of Medicine, National University of Singapore, Singapore, Singapore; 5 Molecular Biology and Lung Cancer Program, Lovelace Respiratory Research Institute, Albuquerque, NM USA

**Keywords:** *ZMYND10*, Breast cancer, *NEDD9*, miR145-5p

## Abstract

**Background:**

Recent studies suggested that *ZMYND10* is a potential tumor suppressor gene in multiple tumor types. However, the mechanism by which *ZMYND10* inhibits breast cancer remains unclear. Here, we investigated the role and mechanism of *ZMYND10* in breast cancer inhibition.

**Results:**

*ZMYND10* was dramatically reduced in multiple breast cancer cell lines and tissues, which was associated with promoter hypermethylation. Ectopic expression of *ZMYND10* in silenced breast cancer cells induced cell apoptosis while suppressed cell growth, cell migration and invasion *in vitro,* and xenograft tumor growth in vivo. Furthermore, molecular mechanism studies indicated that *ZMYND10* enhances expression of miR145-5p, which suppresses the expression of *NEDD9* protein through directly targeting the 3'-untranslated region of *NEDD9* mRNA.

**Conclusions:**

Results from this study show that *ZMYND10* suppresses breast cancer tumorigenicity by inhibiting the miR145-5p/*NEDD9* signaling pathway. This novel discovered signaling pathway may be a valid target for small molecules that might help to develop new therapies to better inhibit the breast cancer metastasis.

## Background

Breast cancer (BC) is the most frequently diagnosed cancer and the leading cause of cancer death among females worldwide, with an estimated 1,762,450 cases and 606,880 deaths in 2019. Breast cancer alone accounts for 30% of all cancer cases and 15% of all cancer deaths among females [1]. In recent years, female breast cancer mortality rates have decreased or remained stable in the western countries, while in China the mortality rates are on the rise [2]. Breast cancer is an extremely heterogeneous disease with varying clinical manifestations and treatment responses [3]. Hence, clinical challenges in the treatment of breast cancer patients remain and it is inevitable that new biomarkers will have to be identified on an individual basis.

*ZMYND10*, also known as *BLU* (zinc finger, MYND-type containing 10), encodes a 50-kD protein containing an MYND-type zinc finger DNA-binding domain in the C-terminus that is commonly found in transcription repressors [4]. *ZMYND10* is located to the 3p21.3 region, and is frequently inactivated or downregulated via genetic or epigenetic changes in many solid tumors, such as lung cancer [5, 6], glioma tumors [7], ovarian cancer [8], liver cancer [9], esophageal squamous cell carcinomas [10], neuroblastoma [11], myelodysplastic syndrome [12], gastric cancer [13], and nasopharyngeal cancer [14]. In recent decades, documented studies have confirmed that *ZMYND10* is a tumor suppressor that can induce apoptosis [8, 15], arrest cell cycle [16], and inhibit proliferation and angiogenesis [17] in different tumors. Some reports have shown that *ZMYND10* can sensitize anticancer activities of chemotherapeutic agents such as gemcitabine [18] and paclitaxel [19]. Although it has been suggested that *ZMYND10* downregulation or silencing is closely correlated to its promoter CpG methylation, its biological functions and molecular mechanisms in breast cancer remain unknown.

*NEDD9* (also known as *HEF1* and *CasL*) is a pro-metastatic gene that is upregulated in different metastatic cancers [20]. It is a cytoplasmic multi-domain scaffolding protein required for mesenchymal invasion and migration driven by extracellular matrix proteolysis. *NEDD9* downregulation has been shown to dramatically reduce cell invasion and metastasis in multiple tumors including breast cancer [21].

In this study, we found that *ZMYND10* suppresses breast cancer tumorigenicity through upregulating miR-145-5p to inhibit the expression of oncogene *NEDD9*, which results in suppression of cell invasion and migration and breast cancer progression.

## Results

### *ZMYND10* downregulation in breast cancer is associated with poor patient survival

**Fig. 1:**
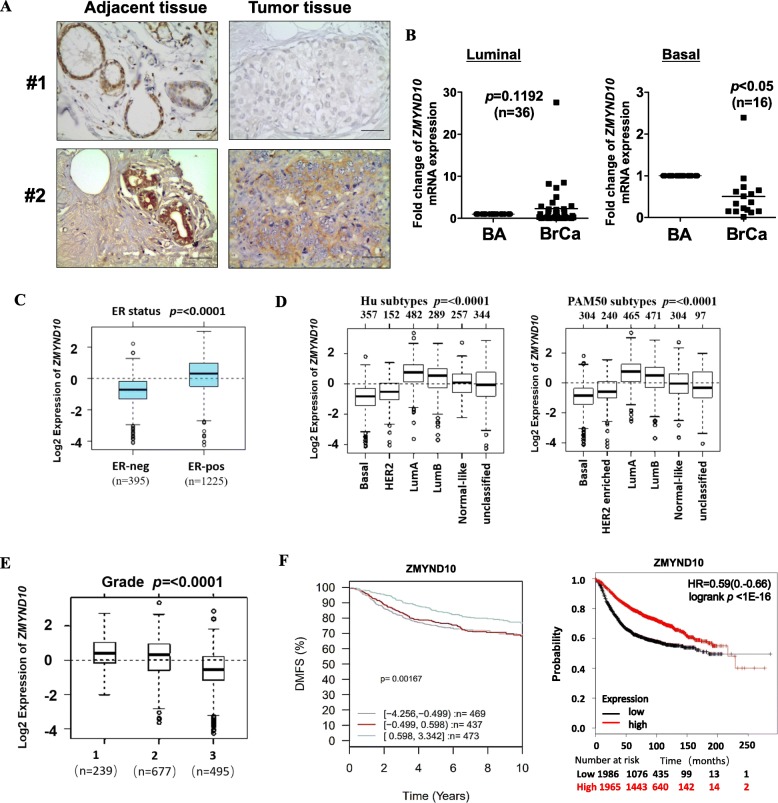
The expression levels of *ZMYND10* in breast cancer tissues. **a** Representative images of *ZMYND10* IHC staining in breast tumor and tumor-adjacent tissues. **b** Quantitative real-time PCR (qPCR) analysis of *ZMYND10* mRNA expression in paired breast tumor and tumor-adjacent tissue samples. **c** Box plot of *ZMYND10* gene expression for tumor samples stratified according to ER status. **d** Box plot of *ZMYND10* gene expression for tumor samples stratified according to Hu subtypes and PAM50 subtypes. **e** Box plot of *ZMYND10* gene expression for tumor samples stratified according to histological grade. **f** Low *ZMYND10* expression is associated with poor 10-year distant metastasis-free survival (DMFS) and relapse-free survival (RFS) in breast cancer patients. Prognosis data was acquired and analyzed using the Gene expression-based Outcome for Breast cancer Online tool (http://co.bmc.lu.se/gobo) and the Kaplan-Meier Plotter database

To investigate whether *ZMYND10* is downregulated in breast cancer, we first used immunohistochemistry assay to examine its expression in tumor-adjacent (*n* = 16) and tumor tissues (*n* = 27). *ZMYND10* expression was significantly lower in breast tumor samples(22/27) than in breast tumor-adjacent tissues (Table 1, Fig. 1a). Furthermore, the *ZMYND10* mRNA expression level was detected by qPCR in paired breast tumor and adjacent non-tumor tissues with different ER/PR/HER2 statuses. *ZMYND10* mRNA levels were much lower in breast cancer tissues than that in normal breast tissue in basal-like (ER-/PR-/HER2-) tumors (14/16). There were no statistical differences in luminal (ER+/PR+/ HER2−or ER+/PR+/ HER2+) tumors (*n* = 36, Fig. 1b). Gene Expression-Based Outcome for Breast Cancer Online (GOBO) (http://co.bmc.lu.se/gobo) database showed consistent results, in which the expression of *ZMYND10* was lower in tri-negative (ER−/PR−/HER2−) tumors compared to that in other molecular type tumors, and was closely related to tumor grade (Fig. 1c–e). Significantly, the prognostic analysis indicated that higher expression of *ZMYND10* was related to better patient survival, which was detected in an integrated database with 3951 cases from the Kaplan-Meier Plotter and in 1379 samples from GOBO (Fig. 1f). Together, these data demonstrated a reduction in *ZMYND10* expression in breast cancer, which may be an indicator of breast cancer prognosis.

**Table 1:** *ZMYND10* protein expression in breast cancer and adjacent tissues

Tissue	Samples	Positive	Negative	*p* value
Breast cancer tissues	27	5	22	0.0181
BC surgical margin tissues	16	9	7	

Note: *BC*, breast cancer

### Promoter methylation of *ZMYND10* contributes to its downregulation in breast cancer

**Fig. 2:**
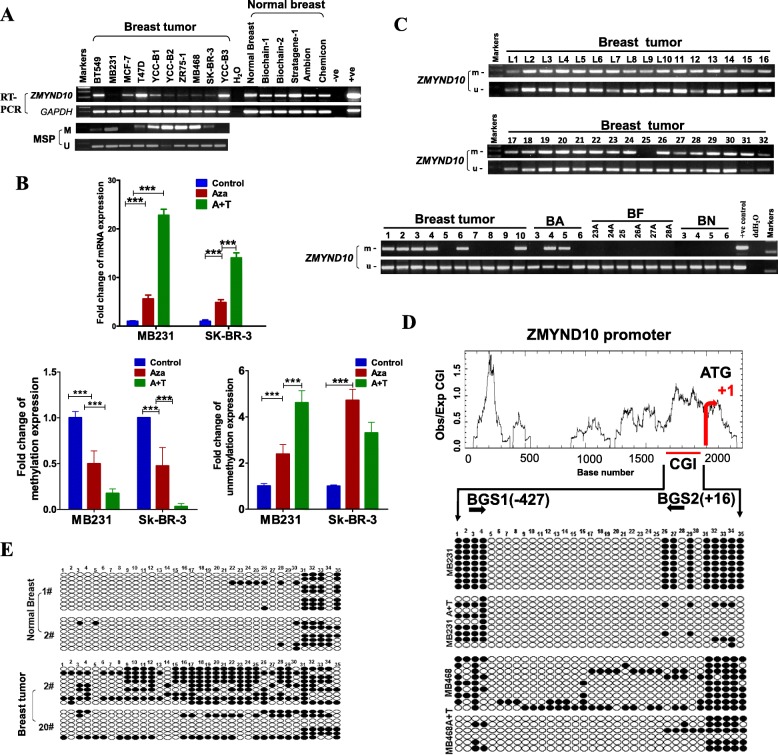
The methylation status of *ZMYND10* promoter in breast carcinoma a RT-PCR and MSP analysis of *ZMYND10* mRNA expression and promoter methylation in breast cancer cell lines. Normal breast tissue samples were used as controls. **b** qPCR indicates demethylation by Aza and TSA (A+T) restored *ZMYND10* expression in MDA-MB231and SK-BR-3 cells. **c** Representative methylation of *ZMYND10* in breast tumor and normal tissues as examined by MSP. **d** Bisulfite genomic sequencing confirmed A+T treatment could inhibit the methylation of the *ZMYND10* promoter. **e** The methylation status of the *ZMYND10* promoter in breast cancer tissues was significantly higher than which in normal breast tissues. Aza, 5-aza-2′-deoxycytidine; BN, breast normal tissue; BF, breast fringe; BA, breast cancer adjacent tissues; M, methylated; U, unmethylated; MSP, methylation-specific polymerase chain reaction; RT-PCR, semiquantitative reverse transcription PCR

DNA methylation is a key mechanism that represses the expression of tumor suppressor genes in cancer. Thus, a possible link between promoter methylation and downregulation of *ZMYND10* expression in breast cancer was investigated. *ZMYND10* was significantly reduced in multiple breast cancer cell lines (7/10), but broadly expressed in all normal breast tissue. MSP analysis showed that *ZMYND10* CpG island was methylated in 80% (8/10) of breast cancer cell lines (Fig. 2a). To further determine whether promoter methylation directly mediates *ZMYND10* silencing, we tested whether *ZMYND10* expression can be restored by pharmacological demethylation in *ZMYND10*-downregulated breast cancer cell lines MDA-MB231 and SK-BR-3 via treating with the DNA methyltransferase inhibitor Aza and histone deacetylase inhibitor TSA. The expression of ZMYND10 was restored after Aza treatment without or with TSA in MDA-MB231 and SK-BR-3 cell lines.

Meanwhile, the results of quantitative methylation-specific PCR (qMSP) showed that the methylation level of *ZMYND10* was decreased and the un-methylation level of *ZMYND10* was increased (Fig. 2b).

MSP was used to examine *ZMYND10* methylation in 138 primary breast cancer tissue, 40 breast cancer-adjacent tissue, 46 breast fringe, and 8 normal breast tissue samples. *ZMYND10* promoter methylation was detected in 101 of 138 (73%) breast cancer tissue samples, but not in normal breast tissues (0/8, Fig. 2c,Table 2). Bisulfite genomic sequencing was then used to measure the methylation of *ZMYND10* CpG in MB468 and MDA-MB231 cells treated with Aza and TSA and two-paired normal breast and breast tumor tissue samples, which verified the MSP results (Fig. 2d, e).

**Table 2:** Methylation status of *ZMYND10* promoter in primary breast tumors

Samples	*ZMYND10* promoter		Frequency of methylation
	Methylation	Unmethylation	
BC (*n* = 138)	101	37	101/138 (73%)
BN (*n* = 8)	0	8	0/8 (0%)

Note: *BC*, breast cancer; *BN*, breast normal *tissues*

The association of *ZMYND10* promoter methylation and patient clinicopathological features was analyzed, which clearly showed that *ZMYND10* methylation was not statistically connected to age, histological type, tumor size, lymph node metastasis, or PR, ER, and HER2 breast cancer patient status (data not shown). These data indicated that *ZMYND10* promoter methylation was common in breast cancer tissues, which is maybe an underlying biomarker for early detection of breast cancer.

### Overexpression of *ZMYND10* inhibited colony formation and proliferation of breast cancer cells

**Fig. 3:**
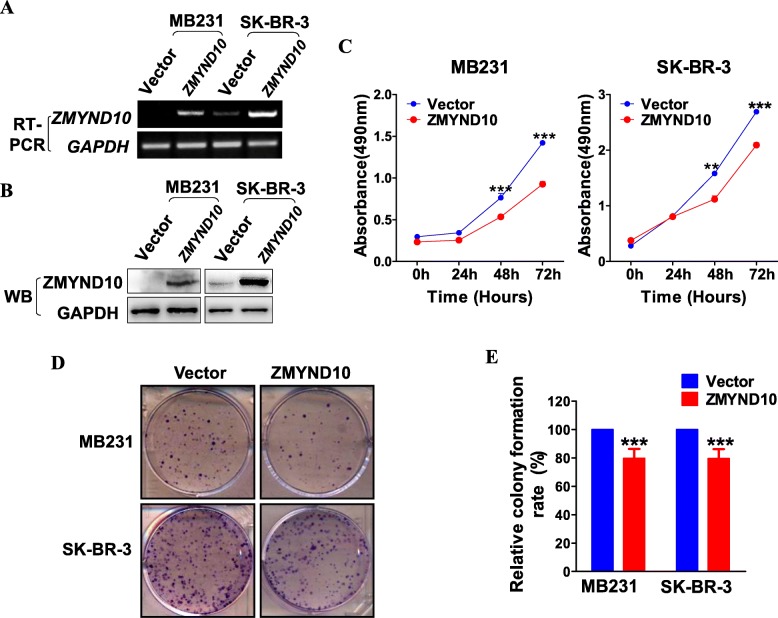
Overexpression of *ZMYND10* inhibited the proliferation of breast cancer cells **a**, **b** Validation of *ZMYND10* overexpression by RT-PCR and Western blot. **c** The capacity of cell proliferation was detected in MDA-MB231 and SK-BR-3 cells stably transfected with *ZMYND10* or empty vector plasmid via MTS assay. **d**, **e** Representative images and the histogram statistics of the colony-formation assay in vector- and ZMYND10-expressed MDA-MB231 and SK-BR-3 cells. Mean ± SD, *p < 0.05, **p < 0.01, ***p < 0.001. All experiments were performed in triplicate, respectively

Silencing of *ZMYND10* by promoter methylation in breast cancer cell lines as well as primary tumors suggested *ZMYND10* as a functional tumor suppressor in breast cancer. Therefore, MDA-MB231(ER−/PR−/HER2−) and SK-BR-3(ER−/PR−/HER2+) cell lines with low expression of *ZMYND10* were selected for a series of functional experiments in vitro. Colony formation and MTS assays were used to evaluate the *ZMYND10* suppressor function. The overexpression of *ZMYND10* in MDA-MB231 and SK-BR-3 cells was detected by RT-PCR and western blot (Fig. 3a, b). When *ZMYND10* was overexpressed, the growth of MDA-MB231 and SK-BR-3 cells was strongly inhibited at 48 and 72 h (*p* < 0.001, Fig. 3c). Smaller and fewer colonies were formed in MDA-MB231 and SK-BR-3 cells expressing *ZMYND10* than that in the empty vector group (*p* < 0.001, Fig. 3d, e).

### *ZMYND10* induces cell cycle arrest in the G2/M phase and promotes apoptosis of breast tumor cells

**Fig. 4:**
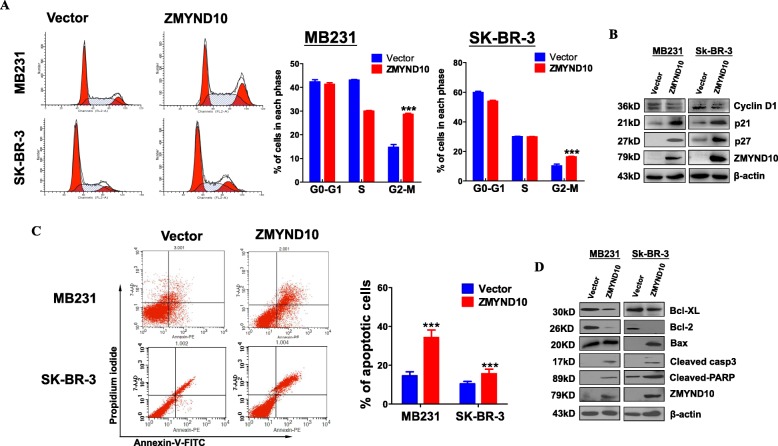
Ectopic *ZMYND10* induced G2/M cell cycle arrest and promote cell apoptosis in breast cancer cells. **a** Flow cytometry analysis was used to detect the effect of *ZMYND10* on cell cycle in vector- and ZMYND10-expressing MDA-MB231 and SK-BR-3 cells. Left, representative flow cytometry plots; right, the histogram statistics of cell cycle changes. **b** Western blotting with antibodies to cell cycle proteins, including cyclin D1, p21, and p27. **c** The proportion of apoptotic cells was detected in vector- and ZMYND10-expressing MDA-MB231 and SK-BR-3 cells by flow cytometry analysis. Representative flow cytometry plots (left) and the histogram statistics of apoptosis changes (right). **d** Western blotting with antibodies to apoptosis proteins, including Bcl-2, Bax, Bcl-xL, cleaved caspase3, and cleaved-PARP

To explore the effects of *ZMYND10* on cell cycle progression, flow cytometry analysis was performed. The results showed that increased cell accumulation in the G2/M phase *ZMYND10*-transfected cells (vector control vs *ZMYND10* = 14.6% vs 28.6% in MDA-MB231 cells, *p* < 0.005; and 10.3% vs 16.3% in SK-BR-3 cells, *p* < 0.005, Fig. 4a). Western blot analysis was used to examine the expression of cell cycle-related proteins. While *ZMYND10* upregulated the expression of protein p27 and protein p21 in MDA-MB231 cells and SK-BR-3 cells, cyclin D1 protein expression was suppressed in *ZMYND10*-transfected cells (Fig. 4b). Annexin V-FITC/PI staining assays were performed to examine apoptosis. Annexin V-PI-positive cells were increased in *ZMYND10*-transfected MDA-MB231 and SK-BR-3 cells to 36.98% and 8.19%, respectively, compared with the controls (*p* < 0.01, Fig. 4c), suggesting that *ZMYND10* can accelerate cell apoptosis. Furthermore, western blot analysis showed that ectopic *ZMYND10* downregulated anti-apoptotic proteins Bcl-xL and Bcl-2 and upregulated the pro-apoptotic protein Bax, cleaved caspase-3, and cleaved PARP in both MDA-MB231 and SK-BR-3 cells (Fig. 4d). These results indicated that *ZMYND10* suppresses cell proliferation through inducing G2/M cell cycle arrest and apoptosis.

### Ectopic *ZMYND10* expression inhibits breast cancer cell migration and invasion

**Fig. 5:**
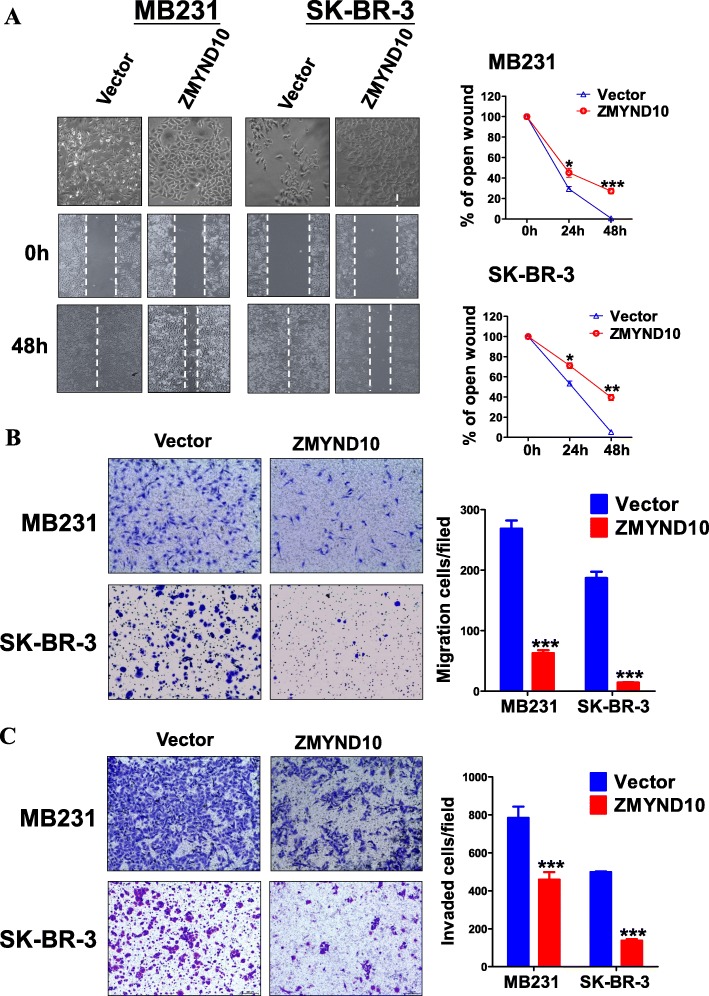
Overexpressed *ZMYND10* suppressed breast cancer cell migration and invasion ability. **a** The cellular migration abilities were examined by wound healing assays in MDA-MB231 and SK-BR-3 stably transfected with *ZMYND10* or empty vector plasmid. Left, photographs were captured at 0 h and 48 h. Right, the ratio of wound healing was calculated. *p < 0.05,**p < 0.01, ***p < 0.001. **b** Representative image(left) and quantification (right) of transwell migration assay conducted with stably transfected MDA-MB231 and SK-BR-3 cells, × 200. **c** Representative image (left) and quantification (right) of transwell invasion assay conducted with stably transfected MDA-MB231 and SK-BR-3 cells, × 200. ***p < 0.001

A wound-healing assay was performed to investigate whether *ZMYND10* suppresses tumor cell migration. Ectopic expression of *ZMYND10* significantly inhibited cell migration from the wound edges compared to the vector control (Fig. 5a). Transwell assay demonstrated a corresponding reduction of migration and invasion in *ZMYND10*-overexpressing cells compared to that in vector-transfected cells (*p* < 0.01, Fig. 5b, c). These results suggested that *ZMYND10* had the capacity to inhibit migration and invasion in vitro.

### *ZMYND10* regulates pathways related to focal adhesion in breast cancer cells

To further study the biological mechanism by which *ZMYND10* suppresses the development of breast cancer, gene expression profiles between the control cells and *ZMYND10*-overexpressing cells were compared using RNA-Sequencing (RNA-Seq). There were 392 differentially expressed genes (DEGs) identified, among which 156 were upregulated and 236 were downregulated (fold change > 2, FDR (false discovery rate) < 0.05, Additional file 1: Figure S1A]. The major identified biological pathways included the focal adhesion, PPAR, and MAPK signaling pathways (Additional file 1: Figure S1B).

### *ZMYND10* inhibits breast cancer by suppressing *NEDD9* expression

**Fig. 6:**
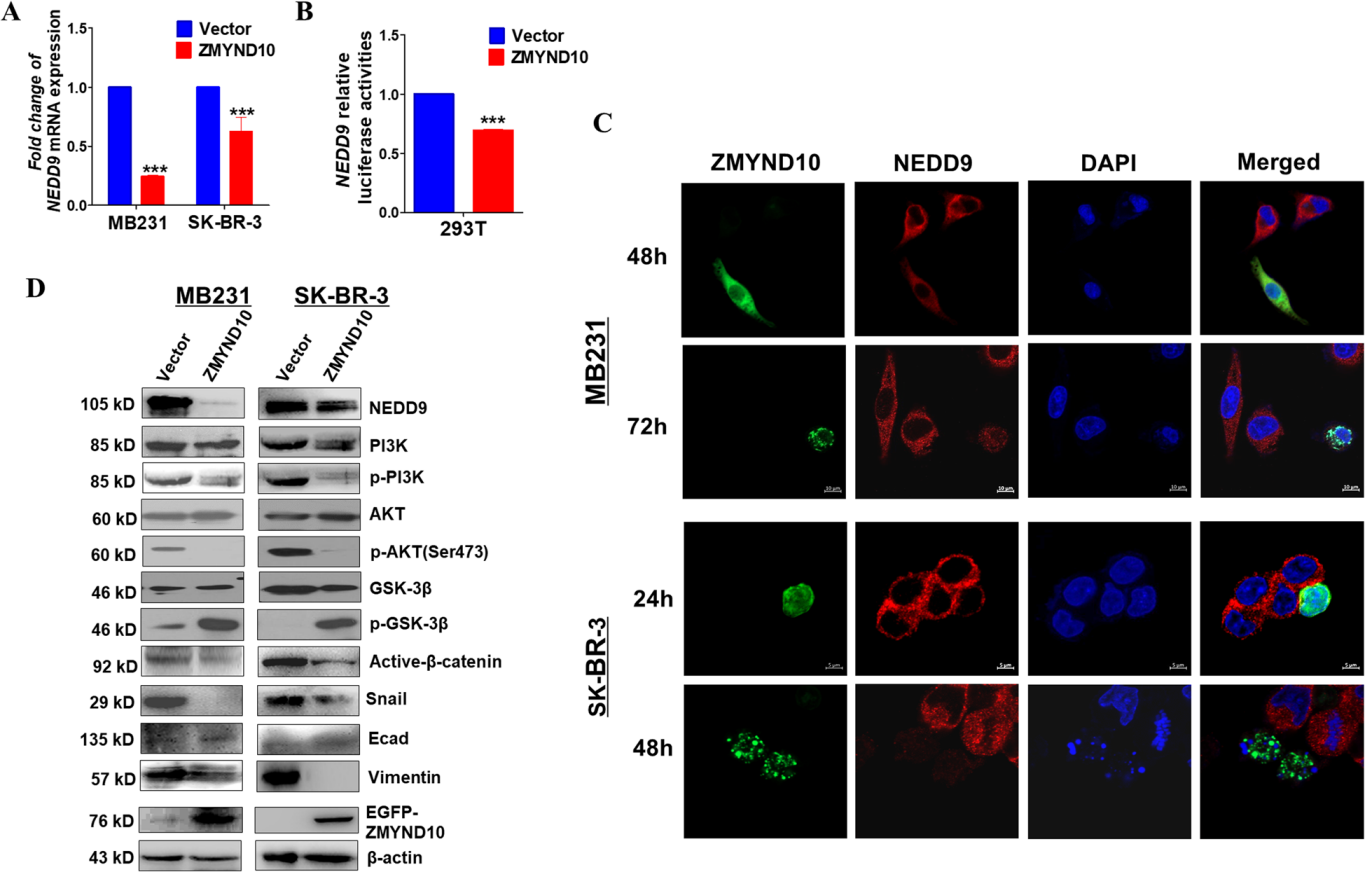
Overexpressed *ZMYND10* inhibited breast cancer metastasis via decreasing the expression of *NEDD9*. **a** The mRNA expression of *NEDD9* was detected by qPCR. **b** Luciferase assay was performed to detect the regulation of *ZMYND10* on *NEDD9* in vector- and ZMYND10-expressed MDA-MB231 and SK-BR-3 cells. **c** Immunofluorescence showed the localization and expression of *NEDD9* in MDA-MB231 and SK-BR-3 cells transfected with ZMYND10 plasmid. **d** After MDA-MB231 and SK-BR-3 cells were overexpressed with ZMYND10, the protein levels of NEDD9, PI3K, p-PI3K, GSK-3β, p-GSK-3β, active-β-catenin, E-cadherin, vimentin, snail, Akt, and the phosphorylation levels of Akt onSer-473 were examined. β-actin was used as an inner control

**Fig. 7:**
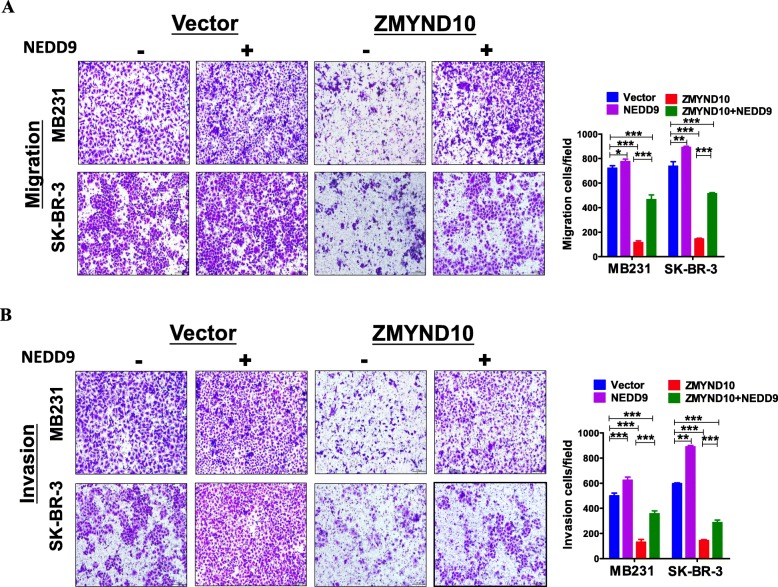
Ectopic *NEDD9* expression partially reversed *ZMYND10*’s effect on migration and invasion. **a** Effect of *NEDD9* overexpression on the migration of MDA-MB231 and SK-BR-3 cells. **b** Effect of *NEDD9* overexpression on the invasion of MDA-MB231 and SK-BR-3 cells. **p < 0.01; ***p < 0.001

Screening of differentially expressed genes in the focal adhesion pathway leads the focus to *NEDD9* that is closely related to breast cancer metastasis (Additional file 1: Figure S1C). qPCR assay was performed to confirm the inhibitory effect of *ZMYND10* on *NEDD9* expression in breast cancer cells (Fig. 6a). And the negative regulatory effect of *ZMYND10* on *NEDD9* was also verified via dual-luciferase reporter assay. These data indicated that *ZMYND10* obviously repressed luciferase reporter activity of *NEDD9* (Fig. 6b). By immunofluorescence detected with laser scanning confocal microscopy, reduced *NEDD9* expression was seen in *ZMYND10*-expressing cells than in the control cells at different time points (Fig. 6c). Because *ZMYND10* was reported to inhibit PI3K/AKT [19] and *NEDD9* participated in AKTactivation in certain circumstances [22], we examined if *ZMYND10* affects this pathway in breast cancer. As expected, *NEDD9,*p-*PI3K,* and *p-AKT* were significantly down-regulated while *p-GSK3β* was markedly upregulated in *ZMYND10*-transfected cells (Fig. 6d). The results clearly showed that *ZMYND10* could inhibit the *PI3K/AKT* pathway in breast cancer cells. Since *NEDD9* belongs to the Cas family of non-catalytic scaffold proteins, it controls cell survival, cell cycle, migration, and adhesion signals. And *NEDD9* was reported to affect the lysosomal degradation of E-cadherin by regulating *SRC* kinase [23], we examined a number of proteins that were involved in EMT (epithelial-mesenchymal transformation ) process. The data showed that *ZMYND10* inhibited the process of EMT. In a rescue experiment, overexpression of *NEDD9* partially attenuated the ability of *ZMYND10* in inhibiting migration and invasion of breast cancer cells (Fig. 7a, b). Altogether, the results suggested that *ZMYND10* is able to suppress migration and invasion of breast cancer cells by inhibiting *NEDD9* expression.

### Mechanism for *ZMYND10* regulation of *NEDD9* expression

**Fig. 8:**
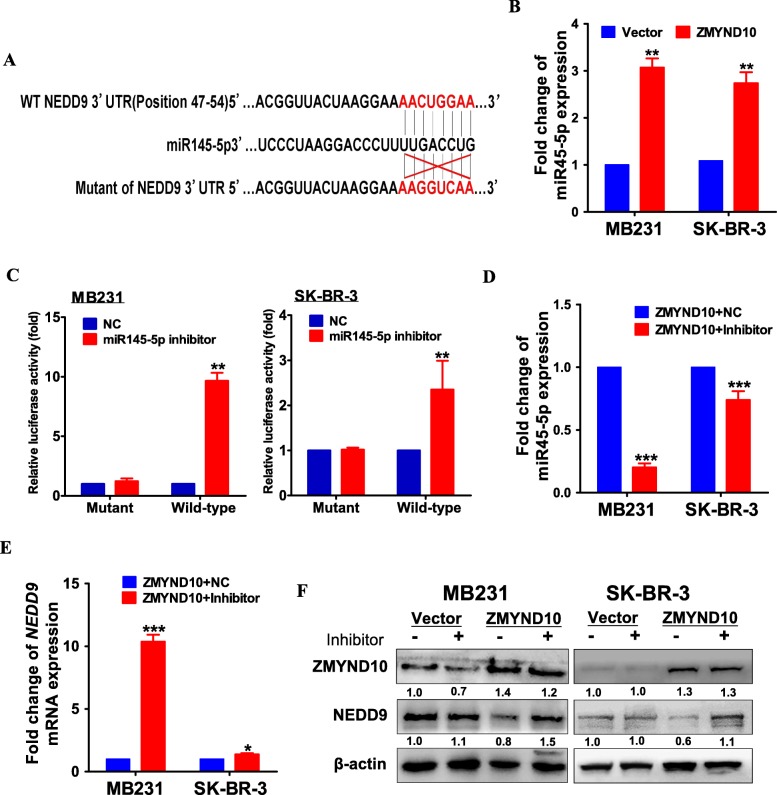
*ZMYND10* decreased the expression of *NEDD9* via altering the miR-145-5p level. **a** Predicted duplex formation between human miR-145-5p and human wild type (WT) NEDD9 3′UTR. **b** The expression of miR145-5p was examined via qPCR in MDA-MB231 and SK-BR-3 cells transfected with vector or ZMYND10 plasmid. **c** Luciferase assay was performed to detect the regulation of miR-145-5p on WT and mutant of NEDD9-3′UTR in MDA-MB231and SK-BR-3 cells transfected with miR-NC or miR145-5p inhibitor oligo. **d** qPCR analysis for miR145-5p expression in MDA-MB231 and SK-BR-3 cells with ZMYND10+miR-NC or ZMYND10+ miR145-5p inhibitor. **e** qPCR analysis for NEDD9 expression in MDA-MB231 and SK-BR-3 cells with ZMYND10+miR-NC or ZMYND10+miR145-5p inhibitor. **f** Western blot assay was performed to detect the protein levels of *NEDD9* in MDA-MB231 and SK-BR-3 cells transfected with p-EGFPc2, p-EGFPc2+ miR145-5p inhibitor, ZMYND10, and ZMYND10+ miR145-5p inhibitor. *p < 0.05;**p < 0.01; ***p < 0.001

Co-immunoprecipitation assay was performed but there was no direct interaction between *ZMYND10* and *NEDD9* detected (data not shown). Thus, we focused on micro-RNA because micro-RNA might play an important role in gene inhibition and activation via a diverse series of mechanisms and may have vital effects on breast cancer progression [24]. Potential micro-RNA binding sites in the 3′-UTR of *NEDD9* were first identified using a bioinformatics website (http://www.targetscan.org/) (Fig. 8), which was consistent with a report in lung cancer [25]. Indeed, *ZMYND10* induced miR145-5p expression in MDA-MB231 and SK-BR-3 cells, which was detected by the qPCR (Fig. 8b). The luciferase reporter assay with the pmiR-RB-Report™ vector carrying MT or WT 3′-UTR sequences of *NEDD9* was used to validate the direct effect of miR-145-5p on *NEDD9* expression. The results showed that the miR-145-5p inhibitor inhibited miR-145-5p expression and induced the activity of WT3′-UTR but not MT3′-UTR reporter (Fig. 8c). The qPCR assay data showed that miR-145-5p inhibitor was added to *ZMYND10*-expressing breast cancer cells could reduce the expression of miR145-5p and increased the expression of *NEDD9* (Fig. 8d, e). Consistent results were also confirmed at the protein level. The miR-145-5p inhibitor enhanced *NEDD9* protein levels and partially reversed *ZMYND10*-decreased *NEDD9* expression (Fig. 8f). Thus, *ZMYND10* may inhibit the expression of *NEDD9* by upregulating miR-145-5p.

### The *ZMYND10*/miR-145-5p axis promotes breast cancer cell migration and invasion by regulating the expression of *NEDD9*

**Fig. 9:**
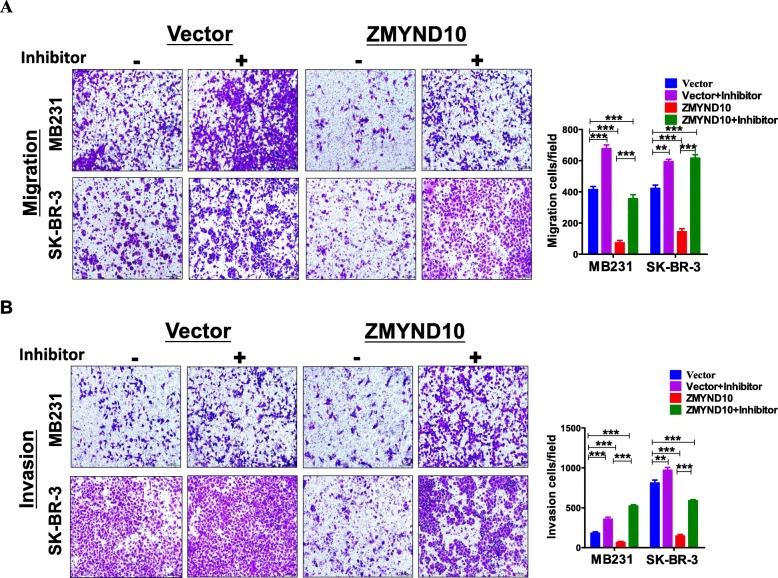
Transfected with miR145-5p inhibitor in MDA-MB231 and SK-BR-3 cell partially reversed *ZMYND10*’s effect on migration and invasion. **a** Effect of miR145-5p inhibitor transfection on the migration of MDA-MB231 and SK-BR-3 cells. **b** Effect of miR145-5p inhibitor transfection on the invasion of MDA-MB231 and SK-BR-3 cells. **p < 0.01; ***p < 0.001

In order to confirm whether miR-145-5p is a key mediator of *ZMYND10* regulating *NEDD9*. We further investigated whether the effect of the *ZMYND10*-miR-145-5p-*NEDD9* signaling axis on migration and invasion of breast cancer was sustained with the miR-145-5p inhibitor. While the miR-145-5p inhibitor enhanced *NEDD9* protein levels and partially reversed *ZMYND10*-decreased *NEDD9* expression, it enhanced cell migration and invasion and attenuated the inhibitory effect of *ZMYND10* decreased on migration and invasion (Fig. 9a, b). These data showed that the *ZMYND10*/miR145-5p/*NEDD9* axis regulates the migration and invasion ability of breast cancer cells, which may contribute to breast cancer metastasis.

### *ZMYND10* inhibits breast cancer growth in vivo

To assess the role of *ZMYND10* in suppressing breast tumor in vivo, a xenograft tumor model was established in nude mice. Tumor size and weight were significantly decreased in tumors derived from *ZMYND10*-expressing MDA-MB231 cells, as compared to mice injected mice with MDA-MB231 cells containing empty vector plasmids (Additional file 1: Figure S2A–C). The immunohistochemical assay showed that the proliferation markers Ki67 and *NEDD9* were significantly reduced in the *ZMYND10*-expressing tumors. (Additional file 1: Figure S2D). These data indicated that *ZMYND10* plays an important role in inhibiting breast cancer in vivo, which is consistent with the previous results in vitro. The above results indicated that *ZMYND10* inhibits migration and invasion of breast cancer by suppressing *NEDD9* expression in vivo and in vitro.

## Discussion and conclusion

Abnormal methylation of tumor suppressor gene promoter CpG islands has been established as a mechanism for transcriptional inactivation of tumor suppressor genes, which is important for the pathogenesis of malignant tumors including breast cancer [26, 27]. *ZMYND10* is contained in a 630-kb region in the 3p21.3, which is frequently and homozygously deleted in multiple cancer types as a result of promoter hypermethylation [28]. This region contains several tumor suppressor genes, such as *RASSF1A*, *CACNA2D2*, *SEMA3B*, and *HYAL1* [5]. Consistent with the literature, *ZMYND10* is an epigenetically regulated tumor suppressor gene and the hypermethylation of its promoter is associated with poor clinical prognosis in several cancer types. However, the function and molecular mechanism of *ZMYND10* in breast cancer are still unknown. Here, we found that *ZMYND10* is downregulated or silenced in breast cancer but not in normal breast and surgical-margin tissues. We also confirmed that promoter hypermethylation of *ZMYND10* is a major cause of its downregulation in breast cancer. Accordingly, *ZMYND10* expression in *ZMYND10*-silenced cells was restored by demethylation treatment. *ZMYND10* repressed breast cancer cell proliferation, promoted G2/M cell cycle arrest, apoptosis, and dramatically lessened migration and invasion of breast cancer cells in vitro. Moreover, *ZMYND10* slowed down the growth of xenograft tumors in vivo. Overall, *ZMYND10* had strong tumor-suppressing effects in breast cancer cells both in vitro and in vivo.

As a potential tumor suppressor gene, *ZMYND10* has been shown to promote apoptosis of tumor cells by regulating sMEK1 activity [15], and inhibition of angiogenesis. However, how *ZMYND10* inhibits tumor metastasis remains unclear. So, we used RNA-sequence analysis to explore how *ZMYND10* performs its cellular functions. The screening results focused on genes and pathways associated with migration and adhesion. Further results indicated that cells overexpressing *ZMYND10* have a poor invasion and migration ability, which confirmed that *ZMYND10* inhibited the invasion and migration of tumor cells. Particularly, we detected that *ZMYND10* repressed the expression of *NEDD9*, a pro-migration protein. Therefore, we concentrated on metastasis to illuminate the tumor inhibition mechanism of *ZMYND10* and identified the miR-145-5p-*NEDD9* signaling pathway downstream of *ZMYND10NEDD9* was found to be closely related to breast cancer metastasis. There is abundant evidence that *NEDD9* is an established marker of metastasis in multiple cancers, including breast cancer [21, 29–38]. *NEDD9* was shown to restore the activity of *MMP14* by promoting the inactivation of Arf6 to facilitate breast cancer metastasis [21]. Other studies also confirmed that *NEDD9* promotes TNBC (triple-negative breast cancer) invasion by regulating the epithelial-mesenchymal transition [39]. However, no relationship has been reported with respect to the roles of *ZMYND10* and *NEDD9* in breast cancer development. We found that *ZMYND10* significantly decreased the expression of *NEDD9*. In addition, restoring *NEDD9* expression facilitated migration and invasion of the *ZMYND10*-expressing cells.

In the process of tumorigenesis and development, the expression of a variety of miRNAs changes, leading to corresponding changes in the expression of downstream target genes and affecting the tumor process [40]. NEDD9 was found to be regulated by miR145-5p by directly targeting the 3′-UTR of *NEDD9*-mRNA in lung cancer [25]. miR145-5p expression was downregulated in breast cancer cells compared to normal human mammary cells, which was reversely correlated to the metastatic ability of breast cancer cells [41]. Therefore, we focused on miR145-5p to interpret the biological mechanism of *ZMYND10* regulating the expression of *NEDD9.* Consistently, we showed that *ZMYND10* downregulates the expression of *NEDD9* through miR145-5p. Although we found that ectopic expression of ZMYND10 increased miR145-5p mRNA expression in MDA-MB231 and SK-BR-3 cells, the mechanism of ZMYND10-mediated miR-145-5p activation has not been fully determined.

Altogether, our study identified *ZMYND10* as a tumor suppressor, which is downregulated in breast cancer due to its promoter hypermethylation, and suggested that *ZMYND10* suppresses breast cancer metastasis by through the miR145-5p/*NEDD9* signaling cascade. Whether this newly identified signaling pathway can be targeted for therapies against breast cancer deserve further studies.

## Methods

### Cell lines, plasmids, and tissue samples

Ten breast cancer cell lines (BT549, MDA-MB231, MCF7, T-47D

YCC-B1, YCC-B2, YCC-B3, ZR-75-1, MB468, SK-BR-3) were kindly provided by Prof. Qian Tao (The Chinese University of Hong Kong). The cells were cultured in the RPMI 1640 medium supplemented with 10% fetal bovine serum (FBS, Gibco-BRL), 100 U/mL penicillin, and 100 mg/mL streptomycin (Gibco-BRL) and incubated in 5% CO_2_ at 37 °C. HEK293T cells were incubated in the DMEM medium (high glucose, HyClone, Logan, USA) with 10% FBS. The pEGFPc2-*ZMYND10* plasmid was constructed by cloning the entire amplified coding region of *ZMYND10* into pEGFPc2 and sequenced for verification. The pEGFPc1-*NEDD9* was constructed in a similar manner. Breast tissues were obtained from the Endocrine and Breast Surgery Department of the First Affiliated Hospital of Chongqing Medical University (Chongqing, China). Every sample was evaluated and subjected to histological diagnosis by expert pathologists. Every patient provided informed consent. Samples were stored at the Chongqing Medical University tissue bank until used in the study. This research was approved by the Institutional Review Board of the Chongqing Medical University(approval notice 20150302).

### Establishing stable cell lines

MDA-MB231 and SK-BR-3 cells were cultured in six-well plates. pEGFPc2-*ZMYND10* or pEGFPc2 was then transfected into 80% confluent MDA-MB231 and SK-BR-3 cells using Lipofectamine 2000 reagent (Lipofectamine 2000 Reagent, Invitrogen, CA, USA) according to the manufacturer’s protocol. After transfection, cells were grown in a non-selective growth medium for 48 h, after which it was replaced with a selection medium containing 24 μL/mL (MDA-MB231) and 10 μL/mL (SK-BR-3) G418 (50 mg/mL) for 14 days. Overexpression of *ZMYND10* was confirmed by western blotting and quantitative real-time PCR (qPCR) before other experimental procedures.

### miRNA inhibitor and transfection

The miR-145-5p inhibitor and negative control miRs (miR-NC) were synthesized by RIBOBIO (Guangzhou Ribobio Co., Ltd). All transfections were implemented using Lipofectamine 2000 according to the manufacturer’s instructions with a concentration of 75 nM miR-145 inhibitor or miR-NC. Total RNA and protein were extracted 48 h post-transfection and used for quantitative real-time PCR (qPCR) and western blot analysis.

### DNA and RNA extraction

Genomic DNA was isolated from BrCa tissues and cell lines using the QIAamp® DNA Mini Kit (Qiagen, Hilden, Germany) and DNAzol® Reagent (Invitrogen) following the manufacturer’s instructions. Total RNA was separated from the BrCa tissues and cell lines using the TRIzol reagent (Invitrogen, Carlsbad, CA, USA). Total RNA and DNA were determined using gel electrophoresis. Samples were reserved at − 80 °C until further use.

### Semiquantitative reverse transcription PCR and qPCR

Reverse transcription was implemented using the Promega GoScript™ reverse transcriptase (Promega). Reverse transcription PCR (RT-PCR) was performed using the Go-Taq (Promega, Madison, WI, USA) and GeneAmp RNA PCR system (Applied Biosystems). β-actin was used as a control. SYBR Green (Thermo Fisher) and 7500 Real-Time PCR System (Applied Biosystems) were used to perform qPCR. GAPDH was amplified as a control. Primer sequences are listed in Table 1.

### 5-Aza-2′-deoxycytidine and trichostatin A treatment

Cells were treated with 10 μM Aza (Sigma-Aldrich, St. Louis, MO, USA) for 72 h and then further treated with 100 nM trichostatin A (TSA) (Cayman Chemical Co, Ann Arbor, MI, USA) for 24 h. RNA was extracted for RT-PCR, and DNA was extracted for methylation-specific PCR(MSP).

### Bisulfite treatment, MSP, and qMSP

Genomic DNA was extracted from tissues and cell lines using the QIAamp DNA Mini Kit (Qiagen, Hilden, Germany). DNA bisulfite treatment was carried out according to previously published methods [42]. MSP primers for *ZMYND10* are listed in Table 3. All primers were previously tested for their inability to amplify unbisulfite DNA. PCR products were analyzed on 2% agarose gels.qMSP was performed as previously described [43].

**Table 3:** List of PCR primers used in this study

PCR	Primer	Sequence (5′–3′)	Product size (bp)	PCR cycles	Annealing temperature (°C)
RT-PCR	*ZMYND10F*	CTCGATATGGGAGACCTG	A.1.1.1.1.1.1.1. 327bp	A.1.1.1.1.1.1.2. 32	A.1.1.1.1.1.1.3. 55
	*ZMYND10R*	CACCACCATGTAGATGGG			
	*NEDD9F*	GCTGGATGGATGACTACGAT	A.1.1.1.1.1.1.4. 145bp		
	*NEDD9R*	AACAGCTGGAACTGGCTCAG			
	*GAPDHF*	GGAGTCAACGGATTTGGT	A.1.1.1.1.1.1.5. 206bp	A.1.1.1.1.1.1.6. 23	
	*GAPDHR*	GTGATGGGATTTCCATTGAT			
qRT-PCR	*ZMYND10F*	CTAACTGAAACCCAGCCTCCTA	A.1.1.1.1.1.1.7. 100bp		A.1.1.1.1.1.1.8. 60
	*ZMYND10R*	TTGCCTGCCACTTGCCTC			
	*miR-145-5pF*	CTGATGGTGGAGAGCTCACA			
	*MiR-145-5pR*	GTGCAGGGTCCGAGGT			
	*MiR-145-5pRT*	GTCGTATCCAGTGCAGGGTCCGAGGTATTCGCACTGGATACGACAGGGAT			
	*U6F*	CTCGCTTCGGCAGCACA			
	*U6R*	AACGCTTCACGAATTTGCGT			
	*U6RT*	AACGCTTCACGAATTTGCGT			
	*GAPDHF*	CCAGCAAGAGCACAAGAGGAA	114bp		
	*GAPDHR*	CAAGGGGTCTACATGGCAACT			
MSP	*ZMYND10m1*	GGTTGTTGTTTAGGATTCGTC	A.1.1.1.1.1.1.9. 178bp	A.1.1.1.1.1.1.10. 40	A.1.1.1.1.1.1.11. 60
	*ZMYND10m2*	AACAATAACTCCGAAACTCCG			
	*ZMYND10u1*	TGGTTGTTGTTTAGGATTTGTT	A.1.1.1.1.1.1.12. 181bp	A.1.1.1.1.1.1.13. 40	A.1.1.1.1.1.1.14. 58
	*ZMYND10u2*	AAACAATAACTCCAAAACTCC A			
BGS	*ZMYND10BGS1*	GGGTAGGTTAAGATGTTATAGT	A.1.1.1.1.1.1.15. 454bp	A.1.1.1.1.1.1.16. 40	A.1.1.1.1.1.1.17. 60
	*ZMYND10BGS2*	AACAACAACAATTCCAAATCTC			

Note: *RT-PCR* semiquantitative reverse transcription PCR, *qPCR* quantitative real-time PCR, *MSP* methylation-specific PCR, *BGS* Bisulfite genomic sequencing

### Colony formation assay

Cells stably expressing *ZMYND10* or vector were plated at a number of different densities in fresh 6-well plates and incubated for 2 weeks with medium containing 10% FBS and G-418. Surviving colonies (≥ 50 cells per colony) were counted after staining with crystal violet. Data were obtained from three independent cultures and each experiment was repeated in three separate wells.

### Cell proliferation assay

Cell proliferation was evaluated with the CellTiter 96 AQueous One Solution Cell Proliferation Assay (MTS, Promega) according to the manufacturer’s instructions. Cells stably expressing *ZMYND10* or vector were seeded in 96-well plates (2000 cells per well) with 200 μL of medium containing 10% FBS and cultured for 24, 48, or 72 h. Cells were then incubated with 100 μL of medium per well containing 20 μL of the CellTiter 96 Aqueous One Solution reagent for 2 h at 37 °C. Absorbance values were measured at 450 nm with the microplate reader (Multiskan MK3, Thermo Fisher Scientific, Schwerte, Germany). Each experiment was repeated three times.

### Flow cytometry analyses of cell cycle and apoptosis

For cell cycle analysis, cells were digested with trypsin and fixed with ice-cold 70% ethanol, treated with 5 mg/mL RNase A (Sigma), stained with propidium iodide, and analyzed by flow cytometry (FACSCalibur instrument and CELLQUEST software, Becton Dickinson). For the apoptosis assays, cells were stained with annexin V-fluorescein isothiocyanate and PI (propidium iodide). Apoptosis and cell cycle status data were analyzed using the CELL Quest software (BD Biosciences, San Jose, CA, USA). All experiments were performed in triplicate.

### Wound healing assay

Cells were seeded in six-well plates and allowed to grow until confluent. Following serum starvation of 24 h, a linear scratch “wound” was created on the cell monolayer with a sterile 10-μL tip. Cells were then washed with PBS (phosphate buffer saline), serum-free media were added, and microscopic cell images (×10 magnification) were collected every 6 h. The linear “wound” was evaluated based on the zero-line. The experiment was performed three times in triplicate.

### Transwell migration and invasion assay

Transwell chamber inserts with 8-μm pores and coated with 70 μL of Matrigel (2.5 mg/mL; BD Biosciences Discovery Labware, 1:7 dilution) were used for the invasion assay. Cells were seeded into the upper wells of pre-coated transwell chambers. Lower wells of the transwell chambers were filled with 700 μL of the same medium with 20% FBS. After a 48-h incubation, cells were fixed with 4% paraformaldehyde for 30 min and then stained for 30 min with Crystal violet. Cells were then wiped off from the upper side of the filter using cotton swabs. Microphotograms of the cells that had migrated to the lower side of the filter were obtained using light microscopy.

### Immunofluorescence

MDA-MB231 and SK-BR-3 cells were seeded on 24-well plates with microcover glass and then transfected with pEGFPc2-*ZMYND10* plasmids for expression of the green fluorescent protein. After 48 h, cells were fixed for 30 min in 4% paraformaldehyde, permeabilized with 0.5% Triton X-100 at room temperature for 10 min, and then blocked with blocking buffer for 1 h. After treatment, the slides were incubated with anti-*HEF1* (*NEDD9*, 1:150; #ab18056; Abcam, USA) at 4 °C. After 20 h, cells were incubated with Alexa Fluor 555-conjugated goat anti-mouse secondary antibodies for 1 h in the dark. All slides were counterstained with 4'-6-diamidino-2-phenylindole (DAPI, Roche, Palo Alto, CA, USA). Photomicrographs were captured with a confocal laser scanning microscope.

### Western blot

Cells were lysed using a protein extraction reagent (Thermo Scientific, Rockford, IL, USA) containing protease inhibitor phenyl methane sulfonyl fluoride and a phosphatase inhibitor cocktail (Sigma, St. Louis, MO, USA). Total protein concentrations were measured using the BCA protein assay reagent (Thermo Scientific, Rockford, IL, USA). Western blot assayswere implemented as previously described [43]. The primary antibodies were used as follows: ZMYND10 (#S0437, Epitomics), p21( #2947,Cell Signaling Technology), p27(#3686, Cell Signaling Technology), Cyclin D1( #sc-20044, , Santa Cruz Biotechnology), Bcl-xL(#2764, Cell Signaling Technology), Bcl-2 (#2870, Cell Signaling Technology), Bax ( #5023, Cell Signaling Technology), cleaved caspase-3 (#9664, Cell Signaling Technology), cleaved PARP(#5625, Cell Signaling Technology), total AKT( #4691, Cell Signaling Technology), Phospho-AKT (#4060,Cell Signaling Technology), total PI3K(sc-423,Santa Cruz Biotechnology), Phosphor-PI3K( #17366, Cell Signaling Technology),total GSK3β( #9315, Cell Signaling Technology), Phospho-GSK3β( #9323, Cell Signaling Technology), Snail ( #3895, Cell Signaling Technology),Active-β-catenin(#19807, Cell Signaling Technology) E-cadherin ( #14472, Cell Signaling Technology),Vimentin ( #5741,Cell Signaling Technology) and NEDD9 (#ab18056, Abcam). β-actin ( #sc-8432, Santa Cruz Biotechnology) served as a loading control. The intensity of the protein bands was gauged with ImageJ 1.52 version(NIH, Bethesda, MD, USA)

### In vivo tumor model

The anti-tumor effect of the target gene was evaluated using an in vivo model. *ZMYND10*-and Vector-expressing MDA-MB231 cells (5 × 10^6^ in 0.2 mL of PBS) were injected subcutaneously into the right and left sides of the back in nude mice, respectively (female, aged 4–6 weeks, weighing 18–22 g, *n* = 4 per group). All procedures for tumor model construction were approved by the Institute Ethics Committee of the First Affiliated Hospital of Chongqing Medical University (approval notice 20150302).

### Immunohistochemistry

Standard streptavidin–peroxidase immunohistochemistry was performed using the UltraSensitive TM SP Kit (Maixin-Bio, Fujian, China) according to the manufacturer’s instructions. Sections were dewaxed, rehydrated and blocked, and then incubated with primary antibodies against ZMYND10 (1:50 dilution, #S0437, Epitomics) and Ki67 (1:100 dilution, #ARG53222, Arigo) at 4 °Covernight. The sections were then treated with a secondary antibody and stained with diaminobenzidine. IHC scores were determined according to previously published methods [44].

### Dual-luciferase reporter assay

To verify *NEDD9* as a direct target of miR-145-5p, target reporter plasmid containing wild-type (WT) and mutant (MT) NEDD9 3′-untranslated region (3′-UTR, Guangzhou Ribobio Co, Ltd., Guangzhou, China) was constructed. MDA-MB231 and SK-BR-3 cells were seeded in 24-well plates and co-transfected with WT or MT reporter plasmid and miR-145-5p inhibitor or miR-NC according to the manufacturer's instructions. Luciferase activity was measured with a dual-luciferase reporter assay kit (Promega) after 48 h. The regulation of *NEDD9* by *ZMYND10* was verified by luciferase reporter assay as previously described [43].

### Statistical analysis

Statistical analyses were performed using GraphPad Prism 5.0 software and IBM SPSS 22.0 software. Two-tailed Student’s *t* tests, the *χ2* test, and Fisher’s exact test were used to evaluate the experimental results. *p* values of all tests were less than 0.05, which was considered statistically significant.

## Supplementary information

**Additional file 1: Figure S1.** RNA-Sequence analysis of *ZMYND10* overexpression in MDA-MB231 cells. (A)The whole distribution of differentially expressed genes in *ZMYND10* stablely transfected MDA-MB231 cells were shown by volcanic map. (B)KEGG pathway classification of differentially expressed genes. The rich factor represents the proportion of differentially expressed genes in specific terms, and the size of the dots represents the number of relevant differentially expressed genes.The Q-value is a calibrated *p*-value. (C)The analysis of differentially expressed genes associated with adhesion is indicated as a heat map. **Figure S2.** *ZMYND10* suppressed xenograft tumor growth in vivo. (A) Image before resection of tumor xenografts. Red round indicated ZMYND10-overexpressing tumors and blue round indicated empty vector control tumors. (B) Image after resection of tumor xenografts. (C) Tumor weight.(D) Representative images of immunohistochemical (IHC) staining. Paraffin sections were stained for *ZMYND10*,Ki67 and *NEDD9,400*×magnification.

## Data Availability

All data supporting the results reported in the article is available from the corresponding author upon a reasonable request.

## Abbreviations

A+T: 5-Aza-2′-deoxycytidine and trichostatin A (TSA)
BA: Breast cancer adjacent tissues
BC: Breast cancer
BF: Breast fringe
BGS: Bisulfite genomic sequencing
BN: Breast normal tissue
DAPI: 4'-6-Diamidino-2-phenylindole
DEGs: Differentially expressed genes
DMFS: Distant-metastasis-free survival
EMT: Epithelial-mesenchymal transition
FBS: Fetal bovine serum
FDR: False discovery rate
GOBO: Gene Expression-Based Outcome for Breast Cancer Online
IHC: Immunohistochemistry
M: Methylated
miRNA: MicroRNA
miR-NC: Negative control miRs
MSP: Methylation-specific polymerase chain reaction
MT: Mutant
PBS: Phosphate-buffered saline
PI: Propidium iodide
qMSP: Quantitative methylation-specific PCR
qPCR: Quantitative real-time PCR
RFS: Relapse-free survival
RNA-Seq: RNA-Sequencing
RT-PCR: Semiquantitative reverse transcription PCR
TNBC: Triple-negative breast cancer
U: Unmethylated
WT: Wild-type
