# Correction to: ZMYND10, an epigenetically regulated tumor suppressor, exerts tumor-suppressive functions via miR145-5p/NEDD9 axis in breast cancer

**DOI:** 10.1186/s13148-022-01256-0

**Published:** 2022-03-09

**Authors:** Yan Wang, Liangying Dan, Qianqian Li, Lili Li, Lan Zhong, Bianfei Shao, Fang Yu, Sanxiu He, Shaorong Tian, Jin He, Qian Xiao, Thomas C. Putti, Xiaoqian He, Yixiao Feng, Yong Lin, Tingxiu Xiang

**Affiliations:** 1 Key Laboratory of Molecular Oncology and Epigenetics, The First Affiliated Hospital of Chongqing Medical University, Chongqing, China; 2 The People’s Hospital of Tongliang District, Chongqing, China; 3 Cancer Epigenetics Laboratory, Department of Clinical Oncology, State Key Laboratory of Translational Oncology, Sir YK Pao Center for Cancer, Li Ka Shing Institute of Health Sciences, The Chinese University of Hong Kong, Hong Kong, Hong Kong; 4 Department of Pathology, Yong Loo Lin School of Medicine, National University of Singapore, Singapore, Singapore; 5 Molecular Biology and Lung Cancer Program, Lovelace Respiratory Research Institute, Albuquerque, NM USA

## Correction to: Clinical Epigenetics (2019) 11:184 10.1186/s13148-019-0785-z

Following the publication of this article, the authors noted that images of SK-BR-3 group in Fig. 9B were misplaced by mistake. The corrected Fig. 9B and corresponding bar graph is now shown in this correction. The authors confirm that the conclusions of this paper are not affected, and sincerely apologize for this error and any inconvenience that may have caused.

**Fig. 9:**
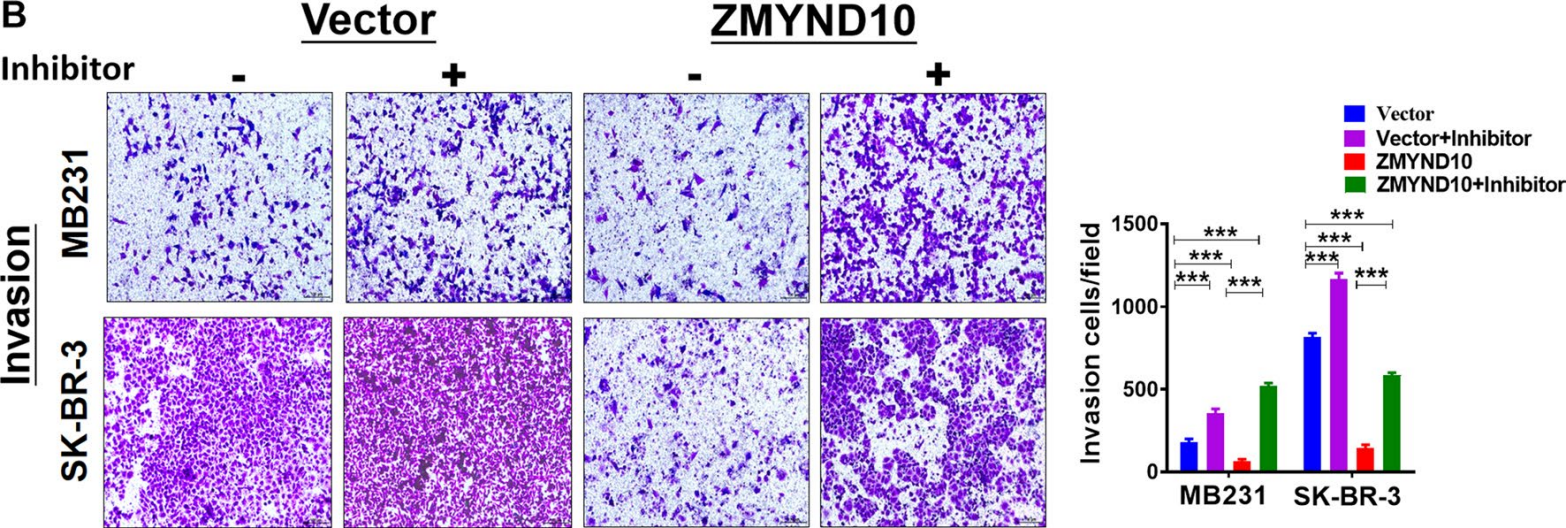
Transfected with miR145-5p inhibitor in MDA-MB231 and SK-BR-3 cell partially reversed ZMYND10’s effect on migration and invasion. **a** Effect of miR145-5p inhibitor transfection on the migration of MDA-MB231 and SK-BR-3 cells. **b** Effect of miR145-5p inhibitor transfection on the invasion of MDA-MB231 and SK-BR-3 cells. **p < 0.01; ***p < 0.001

