# Supplementary figures and images for: ZMYND10, an epigenetically regulated tumor suppressor, exerts tumor-suppressive functions via miR145-5p/NEDD9 axis in breast cancer
Source: Clin Epigenetics. 2019 Dec 4;11:184. doi: 10.1186/s13148-019-0785-z (PMC6894283; doi:10.1186/s13148-019-0785-z)

A

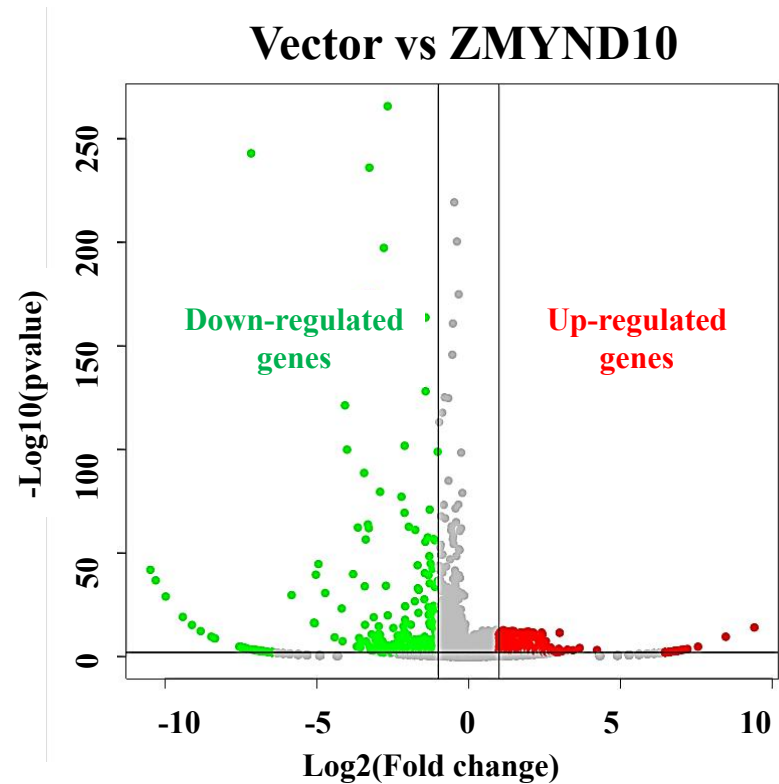

B

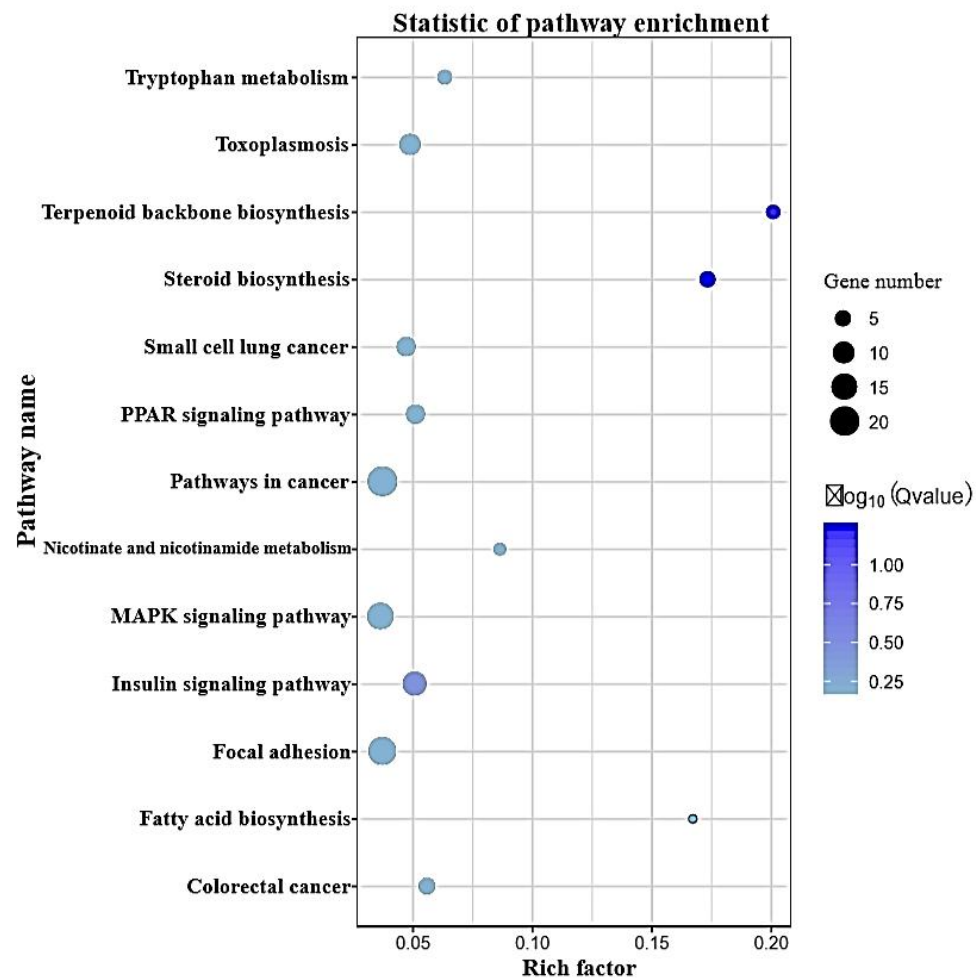

C

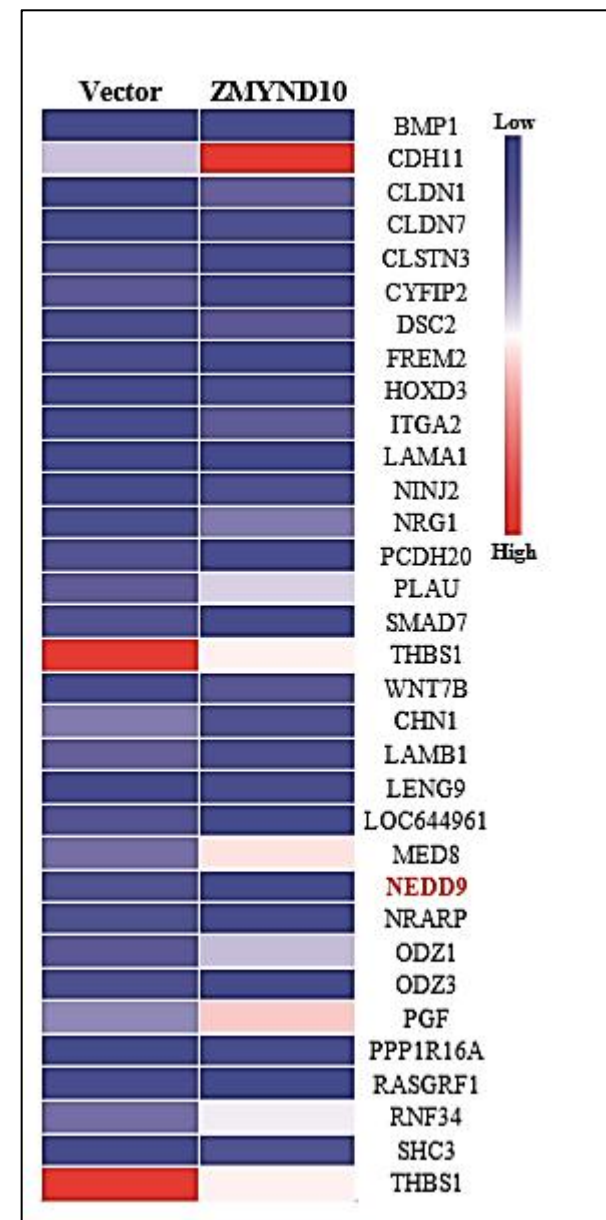

Supplement: Supplementary file 1 — Additional file 1: Figure S1. RNA-Sequence analysis of ZMYND10 overexpression in MDA-MB231 cells. (A)The whole distribution of differentially expressed genes in ZMYND10 stablely transfected MDA-MB231 cells were shown by volcanic map. (B)KEGG pathway classification of differentially expressed genes. The rich factor represents the proportion of differentially expressed genes in specific terms, and the size of the dots represents the number of relevant differentially expressed genes.The Q-value is a calibrated p-value. (C)The analysis of differentially expressed genes associated with adhesion is indicated as a heat map. Figure S2. ZMYND10 suppressed xenograft tumor growth in vivo. (A) Image before resection of tumor xenografts. Red round indicated ZMYND10-overexpressing tumors and blue round indicated empty vector control tumors. (B) Image after resection of tumor xenografts. (C) Tumor weight.(D) Representative images of immunohistochemical (IHC) staining. Paraffin sections were stained for ZMYND10,Ki67 and NEDD9,400×magnification. [file 13148_2019_785_MOESM1_ESM.zip › ZMYND10-BrCa-sfig1.pdf]

A

**MB231**

— ZMYND10

— Vector

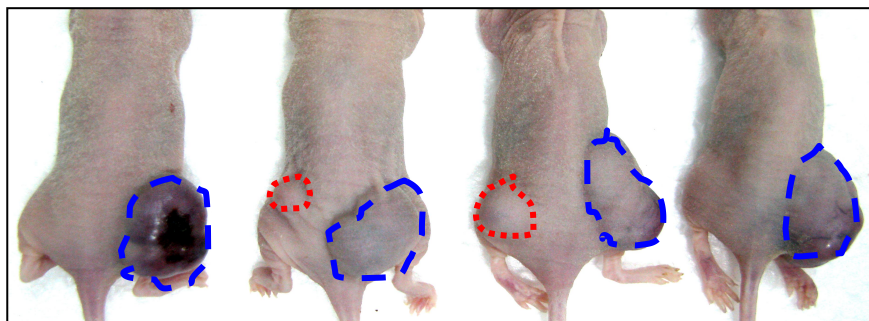

C

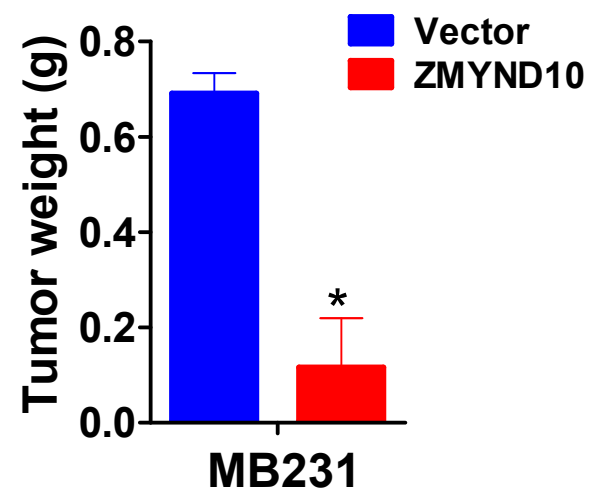

B

**MB231**

Vector

ZMYND10

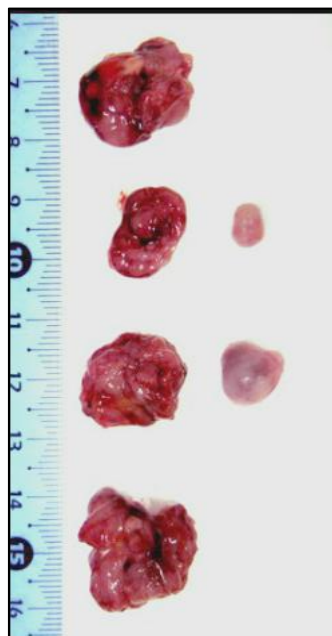

D

**MB231**

Vector

ZMYND10

ZMYND10

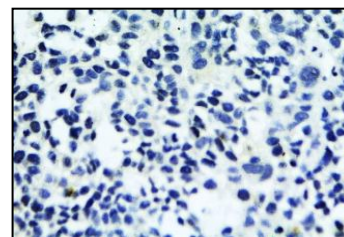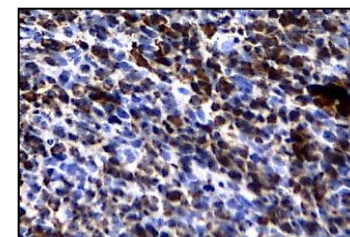

Ki67

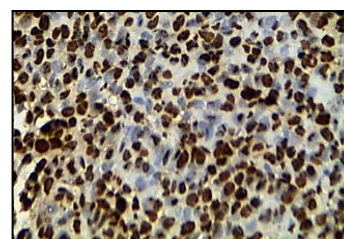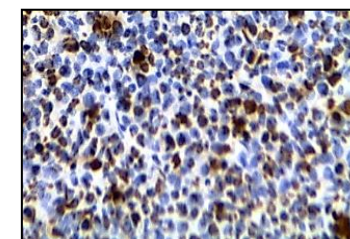

NEDD9

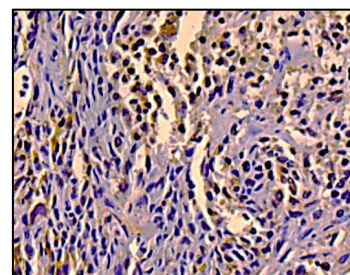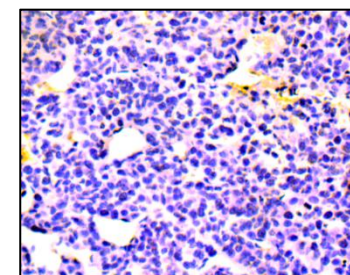

Supplement: Supplementary file 1 — Additional file 1: Figure S1. RNA-Sequence analysis of ZMYND10 overexpression in MDA-MB231 cells. (A)The whole distribution of differentially expressed genes in ZMYND10 stablely transfected MDA-MB231 cells were shown by volcanic map. (B)KEGG pathway classification of differentially expressed genes. The rich factor represents the proportion of differentially expressed genes in specific terms, and the size of the dots represents the number of relevant differentially expressed genes.The Q-value is a calibrated p-value. (C)The analysis of differentially expressed genes associated with adhesion is indicated as a heat map. Figure S2. ZMYND10 suppressed xenograft tumor growth in vivo. (A) Image before resection of tumor xenografts. Red round indicated ZMYND10-overexpressing tumors and blue round indicated empty vector control tumors. (B) Image after resection of tumor xenografts. (C) Tumor weight.(D) Representative images of immunohistochemical (IHC) staining. Paraffin sections were stained for ZMYND10,Ki67 and NEDD9,400×magnification. [file 13148_2019_785_MOESM1_ESM.zip › ZMYND10-BrCa-sFig2.pdf]
